# Effect of heme oxygenase-1 on the differentiation of human myoblasts and the regeneration of murine skeletal muscles after acute and chronic injury

**DOI:** 10.1007/s43440-023-00475-3

**Published:** 2023-03-15

**Authors:** Urszula Głowniak-Kwitek, Asier Laria Caballero, Iwona Bronisz-Budzyńska, Magdalena Kozakowska, Kalina Andrysiak, Jacek Stępniewski, Agnieszka Łoboda, Józef Dulak

**Affiliations:** Department of Medical Biotechnology, Faculty of Biochemistry, Biophysics and Biotechnology, Jagiellonian University, 30-387 Kraków, Poland

**Keywords:** Duchenne muscular dystrophy, HO-1, Induced pluripotent stem cells, *Mdx*, microRNA, miR-206, myomiRs

## Abstract

**Background:**

Impaired muscle regeneration is a hallmark of Duchenne muscular dystrophy (DMD), a neuromuscular disorder caused by mutations in the *DMD* gene encoding dystrophin. The lack of heme oxygenase-1 (HO-1, *Hmox1)*, a known anti-inflammatory and cytoprotective enzyme, was shown to aggravate DMD pathology.

**Methods:**

We evaluated the role of HO-1 overexpression in human induced pluripotent stem cell (hiPSC)-derived skeletal muscle cells (hiPSC-SkM) in vitro and in the regeneration process in vivo in wild-type mice. Furthermore, the effect of cobalt protoporphyrin IX (CoPP), a pharmacological inducer of HO-1 expression, on regeneration markers during myogenic hiPSC differentiation and progression of the dystrophic phenotype was analysed in the *mdx* mouse DMD model.

**Results:**

HO-1 has an impact on hiPSC-SkM generation by decreasing cell fusion capacity and the expression of myogenic regulatory factors and muscle-specific microRNAs (myomiRs). Also, strong induction of HO-1 by CoPP totally abolished hiPSC-SkM differentiation. Injection of HO-1-overexpressing hiPSC-SkM into the cardiotoxin (CTX)-injured muscle of immunodeficient wild-type mice was associated with decreased expression of miR-206 and Myh3 and lower number of regenerating fibers, suggesting some advanced regeneration. However, the very potent induction of HO-1 by CoPP did not exert any protective effect on necrosis, leukocyte infiltration, fibrosis, myofiber regeneration biomarkers, and exercise capacity of *mdx* mice.

**Conclusions:**

In summary, HO-1 inhibits the expression of differentiation markers in human iPSC-derived myoblasts. Although moderate overexpression of HO-1 in the injected myoblast was associated with partially advanced muscle regeneration, the high systemic induction of HO-1 did not improve muscle regeneration. The appropriate threshold of HO-1 expression must be established for the therapeutic effect of HO-1 on muscle regeneration.

**Supplementary Information:**

The online version contains supplementary material available at 10.1007/s43440-023-00475-3.

## Introduction

Skeletal muscles have the unique ability to regenerate in response to acute and chronic injuries. Muscle regeneration involves the time and spatially coordinated interaction between a heterogeneous population of muscle satellite cells (mSCs), interstitial cells, and blood vessels and is mainly controlled by extracellular matrix proteins and secreted factors [1].

In response to injury, mSCs are activated, proliferate, differentiate, and finally fuse, giving rise to newly formed muscle fibers. The differentiation of mSCs is regulated by paired box 7 (Pax7) and the myogenic regulatory factors (MRFs): myogenic regulatory factor 4 (MRF4), myogenic factor 5 (Myf5), myogenic determination 1 (MyoD), and myogenin (MyoG) [2]. mSCs express Pax7, which allows these cells to remain undifferentiated [3]. After muscle injury, a differentiation process is initiated, during which Pax7 expression decreases and the level of Pax7-induced MyoD increases, which then regulates the expression of MyoG and Myf5. This leads to the formation and multiplication of myoblasts [4]. At this stage, cells begin to join to form myotubes and muscle fibers, and the expression of myosin heavy chain (MHC), a marker of differentiated muscle cells, appears [5, 6]. Furthermore, microRNA-mediated modulation of gene expression is necessary for successful myogenesis. In particular, the role of muscle-specific microRNAs, called myomiRs, including miR-1, miR-133a, miR-133b, and miR-206 in the regulation of muscle homeostasis has been highlighted [7, 8].

Several in vivo studies underlined the importance of mSCs in muscle regeneration by showing that mSC ablation results in a prominent impairment of muscle repair machinery [9–11]. In parallel, aggravated differentiation of mSCs can contribute to the pathology of chronic diseases such as Duchenne muscular dystrophy (DMD) [12, 13]. In DMD, repeated cycles of muscle fiber degeneration and regeneration, accompanied by dysregulation of inflammation and continuous infiltration of immune cells, lead to substantial impairment of the regeneration process.

DMD, an inherited X-linked recessive disease caused by more than 7000 patient-specific mutations in the *DMD* gene, resulting in the lack of dystrophin, a structural protein of muscle cells, is still incurable (for references, see: [14]). One of the possible modifiers of disease progression is heme oxygenase-1 (HO-1, *Hmox1/HMOX1*), a cytoprotective enzyme with anti-inflammatory, immunomodulatory and pro-angiogenic properties, that degrades pro-oxidative heme into ferrous ions, biliverdin and carbon monoxide [15]. HO-1 expression is driven, among others, by the nuclear factor erythroid 2-related factor 2 (NRF2) transcription factor and can be easily induced by cobalt protoporphyrin IX (CoPP), the structural analogue of heme [16, 17].

HO-1 has been proposed as a target for DMD treatment. Our previous studies have shown that in a cardiotoxin (CTX)-induced muscle damage model, *Hmox1*^−/−^ knockout mice exhibited augmented muscle damage, altered M1/M2 macrophage proportions, and accelerated rate of myofiber degeneration [18]. Furthermore, dystrophic *mdx* mice with genetic or pharmacological inhibition of HO-1 manifest increased muscle damage and inflammation, along with reduced exercise capacity. Interestingly, although HO-1 level is up-regulated in the muscles of dystrophic mice and in DMD patients, *Hmox1* expression is decreased in mSCs isolated from *mdx* animals, leading to disturbed proliferation and enhanced differentiation patterns, suggesting the importance of HO-1 as a regulator of muscle regeneration mechanisms [13]. We [13] and others [19] have shown that the protective effect of HO-1 on the progression of DMD could be mediated by CO, since treatment with a CO-releasing molecule (CORM) normalized the altered regeneration process in *mdx* SCs and improved the condition of dystrophic muscles. Accordingly, the induction of HO-1 by CoPP attenuated the progression of the disease, as shown, among others, by the decrease in creatine kinase (CK) level, the serum marker of muscle damage [19]. On the other hand, the lack of transcriptional activity of the HO-1 regulator, NRF2, did not have a significant impact on muscle pathology in various models of muscle injury [20].

Therefore, our hypothesis was that HO-1 may regulate muscle regeneration. To shed more light on this aspect, we used two different strategies. In a model of human induced pluripotent stem cells (hiPSCs) differentiated into skeletal muscle cells (hiPSC-SkM), we assessed the effect of HO-1 overexpression on muscle development in vitro and regeneration in vivo. Furthermore, we analysed the impact of HO-1 pharmacological induction by CoPP in the murine model of DMD in vivo. In our hands, HO-1 inhibited some parameters of the myogenic differentiation of hiPSC. However, in experimental pharmacological approach*,* its global induction with CoPP does not affect the pathology of DMD.

## Materials and methods

### Generation and differentiation of human induced pluripotent stem cells and their differentiation into skeletal muscle cells

The control (hiPSC-ctr) and HO-1 overexpressing (hiPSC-HO-1) hiPSC lines were obtained as described in the Supplementary Materials and Methods according to our published protocol [21].hiPSC-ctr and hiPSC-HO-1 were differentiated into skeletal muscle cells (hiPSC-SkM-ctr and hiPSC-SkM-HO-1, respectively) using Skeletal Muscle Differentiation Media (Genea Biocells, currently Amsbio). Briefly, 10,000 cells were seeded in a 24-well plate coated with collagen I (50 µg/ml, CORNING, 354,236) diluted in acetic acid (0.1 M, POCH). In the first step, the Skeletal Muscle Induction Medium was used to induce satellite cell generation. On the eighth day of differentiation, a passage was performed with the seeding of 20,000 cells per well of a 24-well plate coated with collagen I. After day eight of differentiation, the media for induction of myoblast formation (Myoblast Medium) and on day 14, Myotube Fusion Medium were used. The entire procedure took 18–20 days (Suppl. Fig. 1). Cells were grown in an incubator with standard parameters (37 °C, 5% CO_2_, 95% humidity).

To pharmacologically induce HO-1 expression in hiPSC-SkM-ctr cells, the stimulation with 1 µM cobalt protoporphyrin IX (CoPP, Frontier Specialty Chemicals, Co654-9) was performed every 2 days starting from the eighth day of differentiation. The experiment was completed on day 20 of differentiation.

### Animal models

All animal procedures and experiments were performed following national and European legislation and according to the ARRIVE guidelines, after approval by the 2nd Institutional Animal Care and Use Committee (IACUC) in Kraków, Poland (approval number: 59/2018). The animals were kept in standard specific pathogen-free conditions (SPF) with water and food available ad libitum.

To check the effect of hiPSC-SkM on muscle regeneration in vivo, immunodeficient NOD/SCID mice (NOD.CB17*-Prkdc*^*scid*^/NCrCrl, Charles Rivers) were used. The tibialis anterior muscles of an 8–10-week-old NOD/SCID mice were injected with 50 µl of 10 µM CTX (Sigma-Aldrich). After 24 h, 250 000 hiPSC-SkM-ctr and hiPSC-SkM-HO-1 cells on day 13 of differentiation were injected into the injured muscle. The mice were divided into four study groups: mice not injected with CTX (non-treated), mice injected with CTX but without injected cells (control), mice injected with CTX with the administration of hiPSC-SkM-ctr, and mice injected with CTX with the administration of hiPSC-SkM-HO-1. One month after cell administration, bioluminescence imaging was performed. The animals were then sacrificed by CO_2_ exposure and tibialis anterior muscles were collected for downstream analyses (flow cytometry, qRT-PCR, and histology assessment) (Suppl. Fig. 2).

For experiments with CoPP injection, wild-type (WT) (C57BL/10ScSnJ) and *mdx* (C57BL/10ScSn-*Dmd*^*mdx*^/J) mice were used (purchased from Jackson Laboratory, Bar Harbor, ME, USA, stock nos. 000476 and 00101, respectively). Genotyping of animals was performed on DNA extracted from tail biopsies. A group of *mdx* mice (5 weeks of age, n = 4) was treated intraperitoneally with 10 mg/kg of CoPP (dissolved in 0.2 M NaOH and adjusted to pH 7.6) three times a week for 3 weeks and two times in the 4th week. Age-matched *mdx* control mice (n = 3) were injected with vehicle, and WT mice of the same age (n = 4) were left untreated. 24 h after the last injection mice were subjected to treadmill test, and then they were sacrificed by CO_2_ exposure at 9 weeks of age, and subsequently blood, gastrocnemius muscles, and livers were collected for downstream analyses (Suppl. Fig. 3).

### Treadmill test

A treadmill test was performed with an Exer-3/6 (Columbus) treadmill at 15 degrees downhill. Mice were accustomed for the previous 3 days to 15 min sessions at 8 m/min speed. Before the test, the mice were warmed up at 5 m/min speed for 5 min. For the test, the mice ran on the treadmill at 5 m/min for 2 min, 7 m/min for 2 min, 8 m/min for 2 min, and 10 m/min for 5 min. Subsequently, the speed was increased by 1 m/min every min to a final speed of 20 m/min. The treadmill running test was carried out to exhaustion, defined as the inability of the animal to remain on the treadmill despite stimulation by gentle touching.

### Bioluminescence imaging

To assess the presence of hiPSC-SkM-ctr and hiPSC-SkM-HO-1 in NOD/SCID mice, 1 month after the administration of 250,000 cells to CTX-injured muscles, bioluminescence imaging was performed using an IVIS Lumina II detector. 10 min after intraperitoneal injection of luciferin solution (15 mg/ml in PBS, 150 µl/mouse, TriMen Chemicals), mice were anesthetized by isoflurane inhalation (5% v/v isoflurane/air mixture for induction and 1.5–2% v/v isoflurane/air for maintenance of anesthesia) and bioluminescence was recorded in the whole organism and directly in muscles excised from sacrificed animals.

### Cytometric analysis

mSCs were isolated from the tibialis anterior muscle according to our previous experience [13] and were stained with the antibodies that recognize human CD56 (BV 605, BD Horizon, 562,779),$\alpha$7 integrin (PE, R&D Systems, FAB3518P) and CD34 (PerCP-Cy5,5, BD Biosciences, 347,222). Additionally, the GFP signal was analysed. Data were acquired using a Fortessa flow cytometer (BD Biosciences) with FACSDiva software (BD Biosciences).

### Plasma LDH and CK activity measurement

Plasma was obtained from blood collected from the vena cava just before the terminal procedure, by centrifugation at 1000×*g* for 10 min. Lactate dehydrogenase (LDH) and creatine kinase (CK) activity was measured using diagnostic Liquick Cor-CK and Liquick Cor-LDH kits, respectively (Cormay), following the manufacturer’s instructions.

### RNA isolation, reverse transcription, and quantitative real-time PCR (qRT-PCR)

RNA isolation from hiPSC-SkM and from mouse tissues was performed using fenozol (A&A Biotechnology) and QIAzol lysis reagent (Qiagen), respectively, and based on the standard Chomczynski-Sacchi method [22] and phenol–chloroform extraction as described in our previous study [13]. Detailed protocol is provided in the Supplementary Materials and Methods.

### Western blotting

The evaluation of the protein level of HO-1, GAPDH and α-tubulin was performed according to our well-established protocol [13] described in detail in the Supplementary Materials and Methods.

### Histological analyses

The paraffin sections of the gastrocnemius muscles were subjected to hematoxylin and eosin (H&E) and Masson’s trichrome stainings according to the vendor’s instructions (Sigma-Aldrich) and described in the Supplementary Materials and Methods.

### Immunocytochemistry

After removing the medium, the cells in the culture plate were washed with PBS and then fixed with 4% paraformaldehyde (PFA, ChemCruz) for 15 min at RT. After washing with PBS, the cell membranes were permeabilized with 0.05% Triton X-100 (BioShop) in PBS for 3 min at RT. To limit the possibility of nonspecific binding of antibodies, cells were first incubated in a 0.25% glycine solution in PBS (30 min, RT), and then in 3% BSA in PBS (blocking buffer, 1 h, RT). Between each step, cells were washed with PBS. Primary antibodies (anti-MHC, Sigma-Aldrich, M4276; anti-myogenin, Santa Cruz Biotechnology, sc-576; and anti-MyoD, Santa Cruz Biotechnology, sc-304) were diluted 200 times in blocking buffer and incubated overnight at 4 °C. The next day, excess antibodies were washed away and secondary antibodies: goat anti-rabbit IgG Alexa Fluor 568 and goat anti-mouse IgG Alexa Fluor 568 (Thermo Fisher Scientific, A11011 and A11004, respectively, dilution 1:400) were administered for 2 h in the dark at RT. The nuclei were stained with Hoechst 33,342 (2 µg/ml, Sigma-Aldrich). The staining was analysed under a Nikon Eclipse fluorescence microscope.

### Immunohistofluorescence staining

The frozen sections were used to assess necrosis content (accumulation of IgG, IgA, and IgM antibodies) according to our previous studies [13] and using the detailed protocol provided in the Supplementary Materials and Methods.

### Statistical analysis

Data are presented as mean ± SEM. Statistical analysis was performed using GraphPad Prism by a one-way ANOVA followed by Tukey's post hoc test, and, when indicated, the unpaired 2-tailed Student’s t-test or the Kruskal–Wallis test. The Shapiro–Wilk tested data normality and Grubb’s test was used to identify statistically significant outliers. Differences were accepted as statistically significant for *p* < 0.05. The n-number indicating the number of experimental repetitions or animals per group, as well as the specific statistical test used, were included in the figure legend.

## Results

### Establishment of hiPSC with overexpression of HO-1 and their differentiation into skeletal muscle cells

**Fig. 1:**
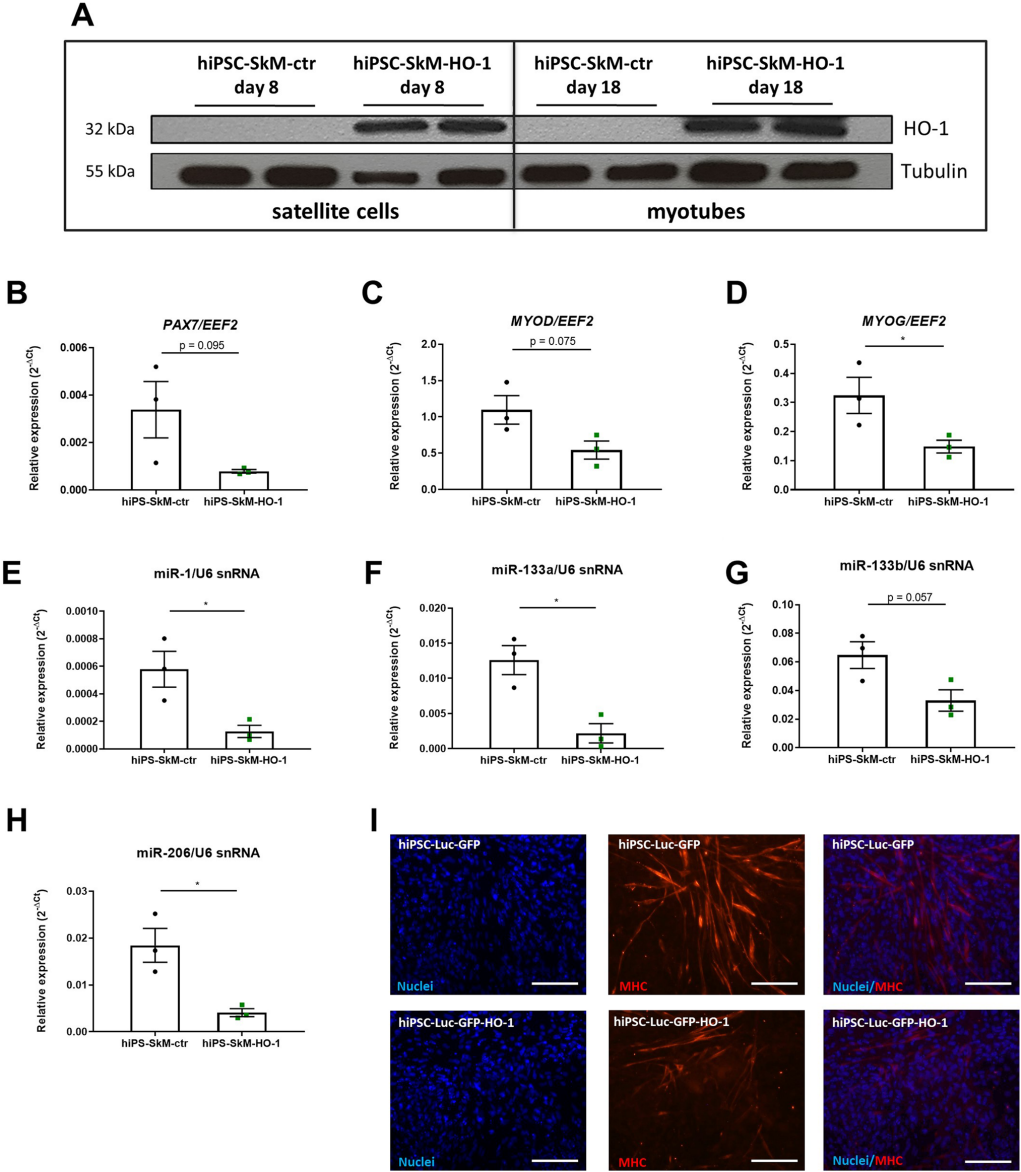
Overexpression of heme oxygenase-1 (HO-1) in hiPSC-derived muscle cells affects cell differentiation. **A** Western blot analysis of HO-1 expression at 8 and 18 days of hiPSC-ctr and hiPSC-HO-1 differentiation into skeletal muscle cells. **B**–**H** Gene expression on day 15 of myogenic differentiation of hiPSC-SkM-ctr and hiPSC-SkM-HO-1. qRT-PCR analysis of **B** *PAX7* (paired box 7), **C** *MYOD* (myogenic determination 1), **D** *MYOG* (myogenin), **E** miR-1, **F** miR-133a, **G** miR-133b, and **H** miR-206. Results are shown as mean ± SEM, *n* = 3, *p < 0.05; unpaired 2-tailed Student’s t-test. **I** Expression of myosin heavy chain (MHC) assessed by the immunofluorescence staining, day 18 of differentiation. Representative photos from fluorescence microscopy, scale bar = 100 μm (200 × magnification), blue: cell nuclei, red: MHC

To evaluate the role of HO-1 in the differentiation of human mSCs, hiPSCs were transduced with lentiviral vectors containing reporter genes – luciferase and GFP (control cell line-hiPSC-ctr) or reporter genes and HO-1 (cell line overexpressing HO-1-hiPSC-HO-1) as previously described [21]. The differentiation toward skeletal muscle cells was performed using a commercially available Skeletal Muscle Differentiation kit, and this three-step method required a specific media change and cell passaging (Suppl. Fig. 1). Western blot performed on protein lysates from satellite cells (day 8 of differentiation) and myotubes (day 18 of differentiation) indicated a high level of HO-1 protein in hiPSC-SkM-HO-1 cells compared to the hiPSC-SkM-ctr cell line (Fig. 1A).

The differentiation of hiPSCs into satellite cells and then into skeletal muscle cells requires the activation of many muscle-specific transcription factors, such as Pax7, MyoD, and myogenin, respectively. When the expression of these factors was analyzed on 15 day of differentiation by qRT-PCR, the tendency for decreased PAX7 (Fig. 1B) and *MYOD* (Fig. 1C) was observed, while the significantly lower level of *MYOG* (Fig. 1D) was found in hiPSC-SkM-HO-1 cells compared to the hiPSC-SkM-ctr cell line (p = 0.0416).

Several miRNAs also play an important role in the process of myogenesis. The expression of muscle-specific miRNAs: miR-1, -133a, -133b, and 206 increased together with the progression of differentiation, and was at the highest level on day 15 of differentiation when cells fuse to form myotubes (data not shown). Importantly, the expression of miR-1 (p = 0.0304), miR-133a (p = 0.0104), miR-133b (p = 0.0570), and miR-206 (p = 0.0108)declined in the HO-1-overexpressing line at the myotube stage compared to normal cells (Fig. 1E, H).

In addition, we checked MHC protein level in cells in the further stage of differentiation (day 18). The hiPSC-SkM-HO-1 line showed a lower level of this mature myogenic marker compared to the control line (Fig. 1I).

### The effect of transplantation of hiPSC-SkM overexpressing HO-1 on muscle regeneration in CTX-induced muscle injury

In order to evaluate whether the observed effects in vitro will affect the behaviour of cells in vivo, hiPSC-SkM cells from day 13 of differentiation were injected into the CTX-injured tibialis anterior muscle of immunodeficient NOD/SCID mice. After 1 month of cell delivery, we checked their presence in the tibialis anterior muscles by measuring luciferase activity with the IVIS Lumina II detector system. Analysis of tibialis anterior muscles, both in vivo and ex vivo after isolation, revealed no bioluminescence signal in any of the studied groups (data not shown). The IVIS results were also confirmed by cytometric analysis demonstrating the absence of GFP-positive cells in the muscles of mice injected with human cells **(**Suppl. Fig. 4A). Similarly, flow cytometry did not show the presence of human CD56 and α7integrin positive cells in mouse muscles (Suppl. Fig. 4B).

**Fig. 2:**
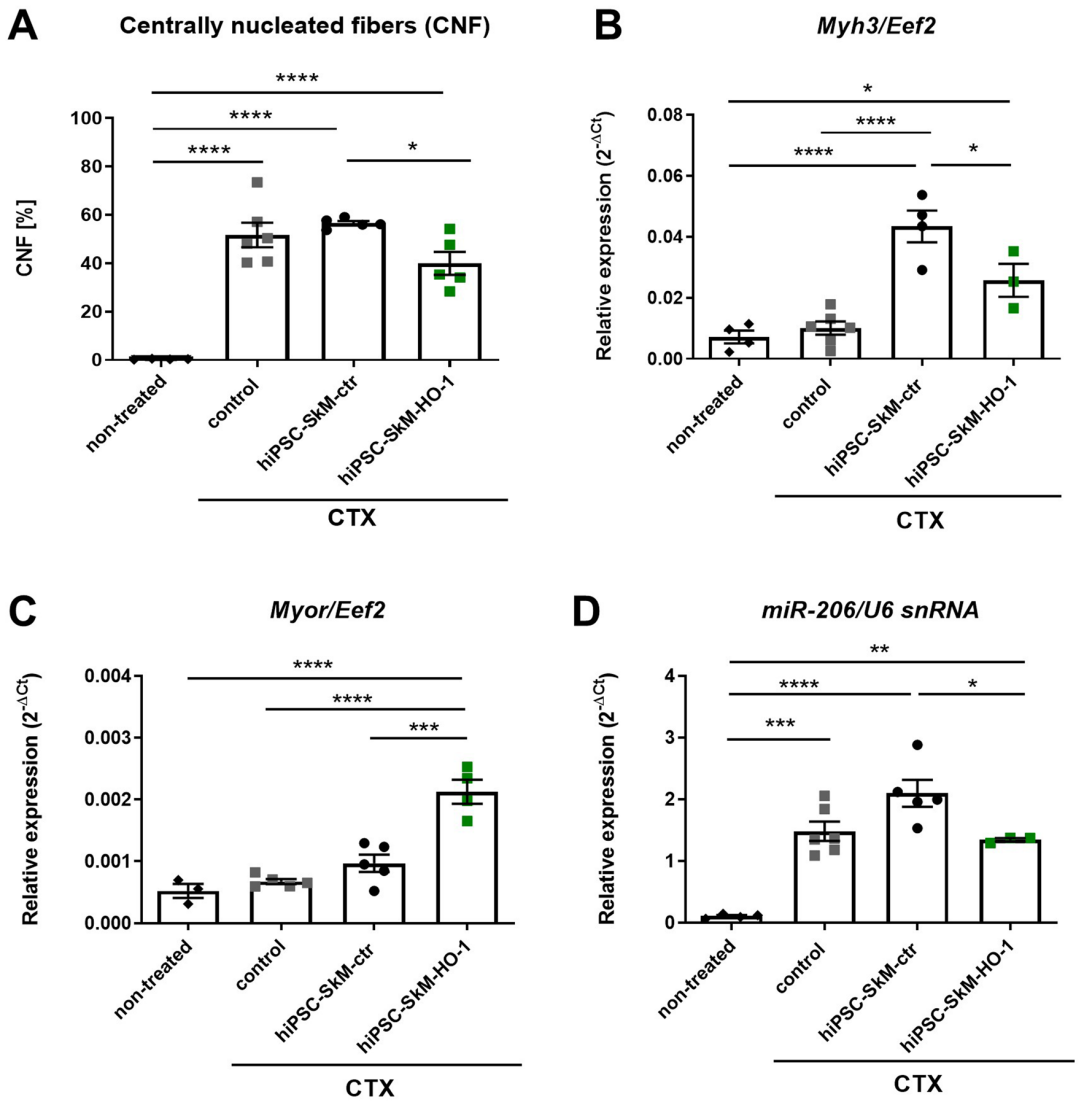
Transplantation of heme oxygenase-1 (HO-1) overexpressing hiPSC-SkM results in decreased regenerative potential. **A** Semiquantitative analysis of centrally nucleated fibers (CNF) performed based on hematoxylin and eosin staining showing a high increase after cardiotoxin (CTX) delivery and the inhibitory effect of HO-1 overexpression. **B**–**D** Gene expression in tibialis anterior muscles isolated from NOD/SCID mice: **B** *Myh3* (embryonic myosin heavy chain), **C** *Myor* (myogenic repressor), **D** miR-206. Results are shown as a mean ± SEM, *n* = 3–6, *p < 0.05; **p < 0.01; ***p < 0.05; ****p < 0.001; a one-way ANOVA followed by Tukey’s post-hoc test

The lack of hiPSC-SkM detection 1 month after in vivo delivery into injured muscle did not exclude their possible effect on the earlier stages of the regeneration process. Therefore, we performed further studies to evaluate the influence of hiPSC-SkM transplantation on the regenerative potential. First, we counted the number of centrally nucleated fibers, which are markers of muscle regeneration. A potent increase in the number of fibers with centrally located nucleus was found in all mice injected with CTX. Importantly, a one-way ANOVA detected statistically significant differences in the percentage of regenerating fibers between analyzed groups (*F*_3, 16_  = 36.63, *p* < 0.0001). In animals injected with cells overexpressing HO-1, the number of centrally nucleated fibers was reduced compared to hiPSC-SkM-ctr cells (*p* = 0.0392) **(**Fig. 2A). Additionally, the expression of the embryonic myosin heavy chain isoform *Myh3*, which encodes eMHC, especially relevant in muscle regeneration [23], also decreased in animals injected with cells overexpressing HO-1 (*p* = 0.0332) (Fig. 2B). Finally, when a one-way ANOVA was applied to test the differences between *Myor,* a myogenesis repressor, and miR-206 in the experimental setup, a significant effects were revealed (*F*_3, 13_ = 28.05, *p* < 0.0001 and *F*_3, 14_  = 24.83, *p* < 0,0001, respectively). Specifically, a potent increase in *Myor* level (*p* = 0.0001) (Fig. 2C) and a statistically significant decrease in miR-206 expression (*p* = 0.0465) (Fig. 2D) were observed after administration of hiPSC-SkM-HO-1 cells compared to control counterparts.

### Stimulation with CoPP up-regulates HO-1 and decreases MHC levels in hiPSC-skeletal muscle cells

**Fig. 3:**
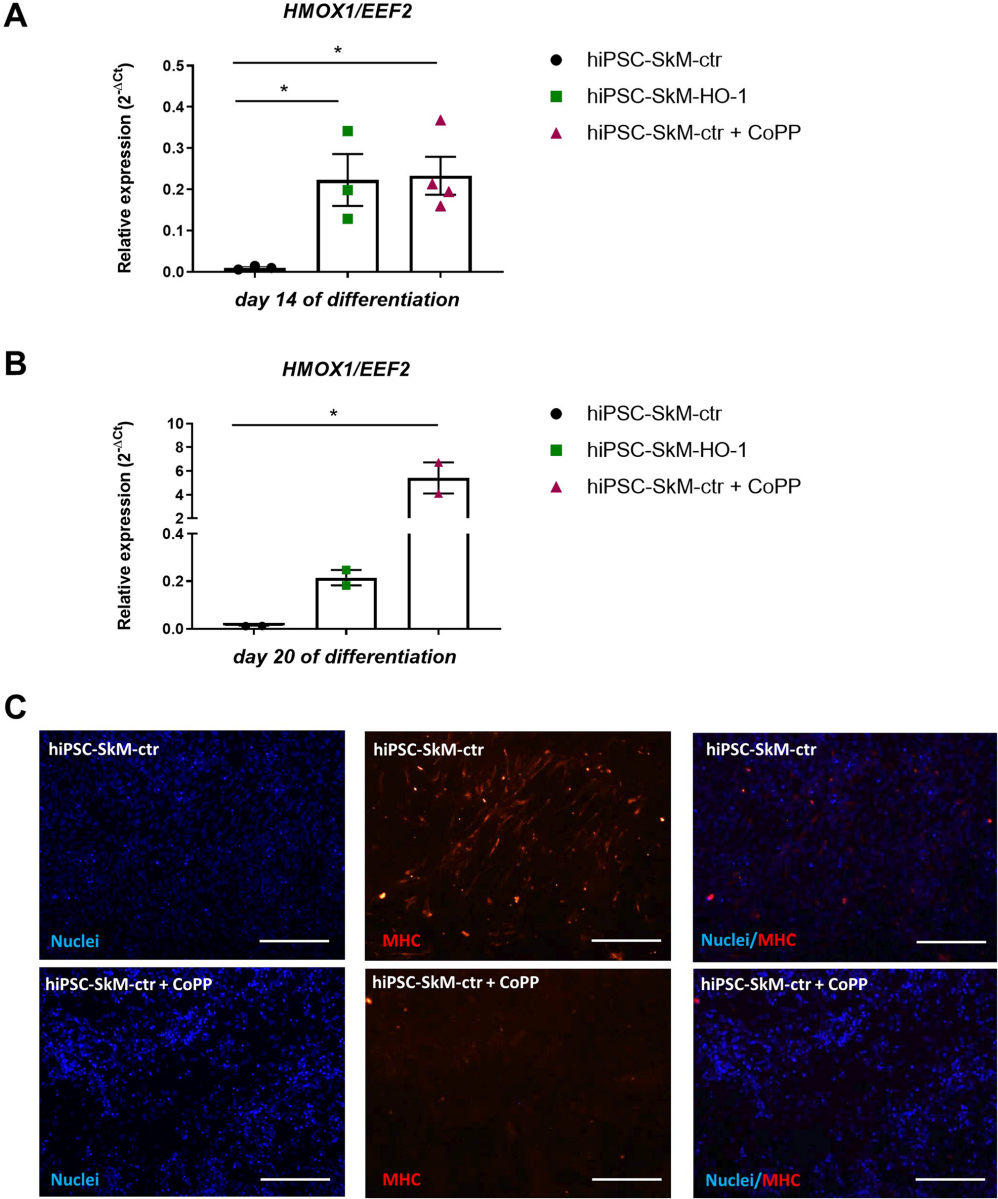
Differentiation of hiPSCs towards skeletal muscle cells after cobalt protoporphyrin (CoPP) stimulation. Expression of the *HMOX1* gene in hiPSC-SkM-ctr, hiPSC-SkM-HO-1 and hiPSC-SkM-ctr stimulated with 1 µM CoPP at days 14 (**A**) and 20 (**B**) of differentiation, qRT-PCR analysis. Results are shown as a mean ± SEM, *n* = 2–4, *p < 0.05; a one-way ANOVA followed by Tukey’s post-hoc test (**A**); the Kruskal–Wallis test (**B**). **C** Expression of myosin heavy chain (MHC) assessed by the immunofluorescence staining, day 20 of differentiation of hiPSC-SkM-ctr and CoPP-treated cells (hiPSC-SkM-ctr + CoPP), fluorescence microscopy, scale bar = 100 μm (100 × magnification), blue: cell nuclei, red: MHC

To further confirm that the observed changes in the expression of myogenic factors between differentiating hiPSC-SkM-ctr and hiPSC-SkM-HO-1 lines depend on HO-1 overexpression, we analysed the effect of pharmacological induction of HO-1 using cobalt protoporphyrin IX (CoPP) on to myogenic differentiation of control cells. In particular, from day eight, hiPSC-SkM-ctr cells were stimulated every 2 days with 1 µM CoPP until day 20 of differentiation. CoPP treatment did not affect cell morphology at any analysed time-point **(**Suppl. Fig. 5). qRT-PCR confirmed a strong and comparable induction of HO-1 expression between hiPSC-SkM-HO-1 and CoPP-stimulated hiPSC-SkM-ctr on day 14 (Fig. 3A), while on day 20 of differentiation approximately 27 times higher induction by CoPP was found compared to genetic overexpression of HO-1 (Fig. 3B). Importantly, on day 20 of differentiation, immunofluorescence staining revealed the presence of MHC in hiPSC-SkM-ctr, but it was not detected in myoblasts treated with CoPP (Fig. 3C). This indicates that strong overexpression of HO-1 induced by CoPP totally inhibits the maturation of skeletal muscle cells (Fig. 3C).

### CoPP treatment leads to an upregulated expression of HO-1 in mdx mice

**Fig. 4:**
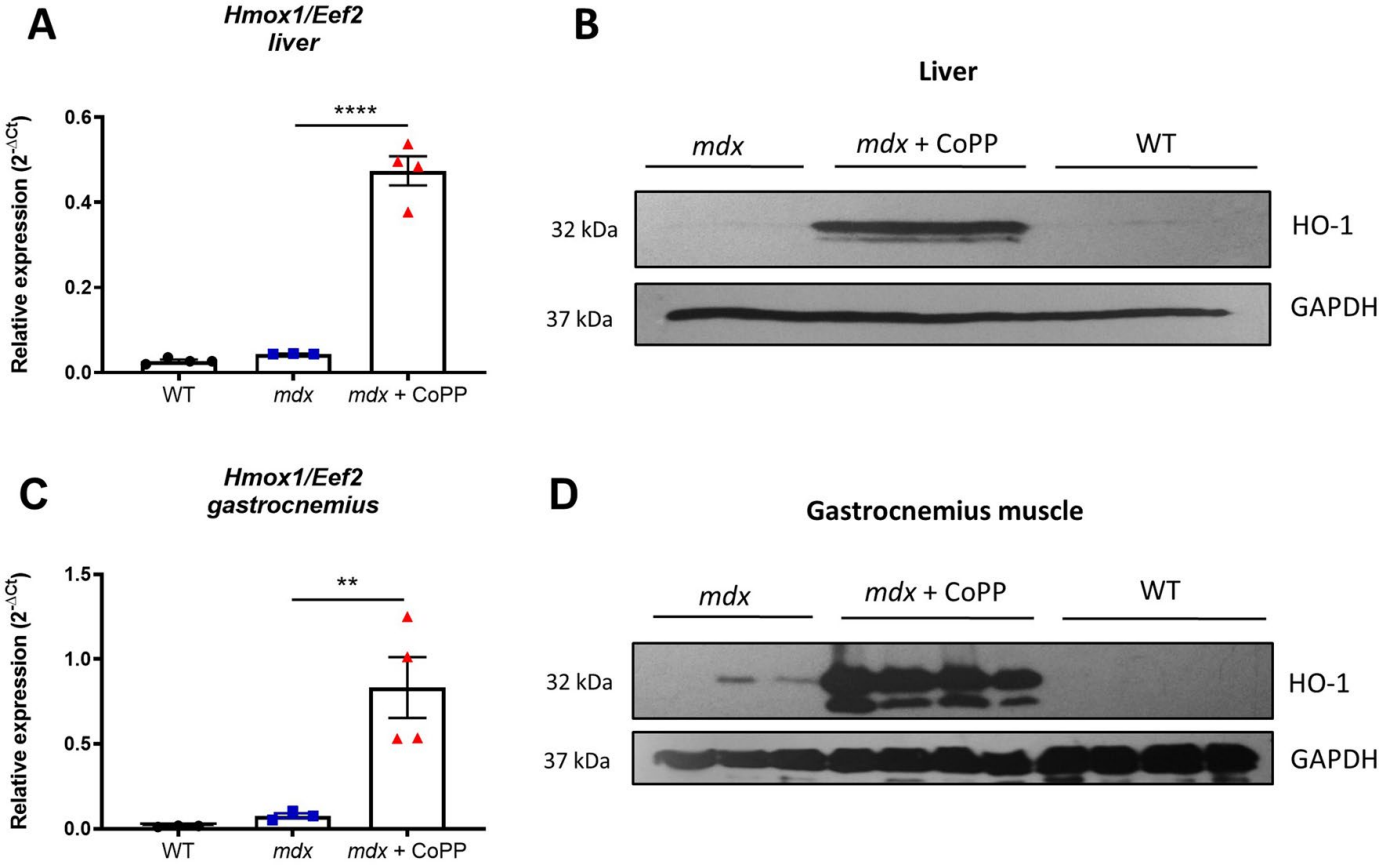
Cobalt protoporhyrin (CoPP) treatment leads to an up-regulated expression of heme oxygenase-1 (HO-1) in *mdx* mice. Dystrophic animals at 5 weeks of age were treated intraperitoneally with 10 mg/kg of CoPP three times a week for 3 weeks and two times in the 4th week, while age-matched *mdx* control mice were injected with vehicle, and WT mice of the same age were left untreated. At the end of the experiment, the expression of *Hmox1*/HO-1 in the liver and gastrocnemius muscle was analysed. **A**, **C** Relative expression of the *Hmox1* gene in the liver and gastrocnemius muscle, respectively. Results are shown as a mean ± SEM, *n* = 3–4, **p < 0.01, ****p < 0.0001; a one-way ANOVA followed by Tukey’s post-hoc test, real-time PCR. **B**, **D** Expression of the HO-1 protein in the liver and gastrocnemius muscle, respectively; *n* = 3–4, Western blotting

To further evaluate the role of CoPP-induced HO-1 in muscle regeneration, we performed in vivo studies on the *mdx* mice, a model of DMD. To confirm the effectivity of CoPP as an inducer of HO-1 in vivo, qRT-PCR and Western blotting were performed to assess the expression of HO-1 at both the mRNA and protein levels. A one-way ANOVA for each group showed a significant effect of CoPP on the *Hmox1* expression in the liver (*F*_2, 8_ = 139.9, *p* < 0.0001) and gastrocnemius muscle (*F*_2, 7_ = 13.5, *p* = 0.0040). On the mRNA level, much stronger induction of *Hmox1* gene expression was observed in the liver (p < 0.0001) than in skeletal muscle (gastrocnemius) (*p* < 0.0097) (Fig. 4A, C), but in both tissues it was very high, and confirmed on the protein level as shown by Western blotting (Fig. 4B, D).

### Muscle functionality, inflammation, and degeneration do not change after CoPP treatment in dystrophic mice

**Fig. 5:**
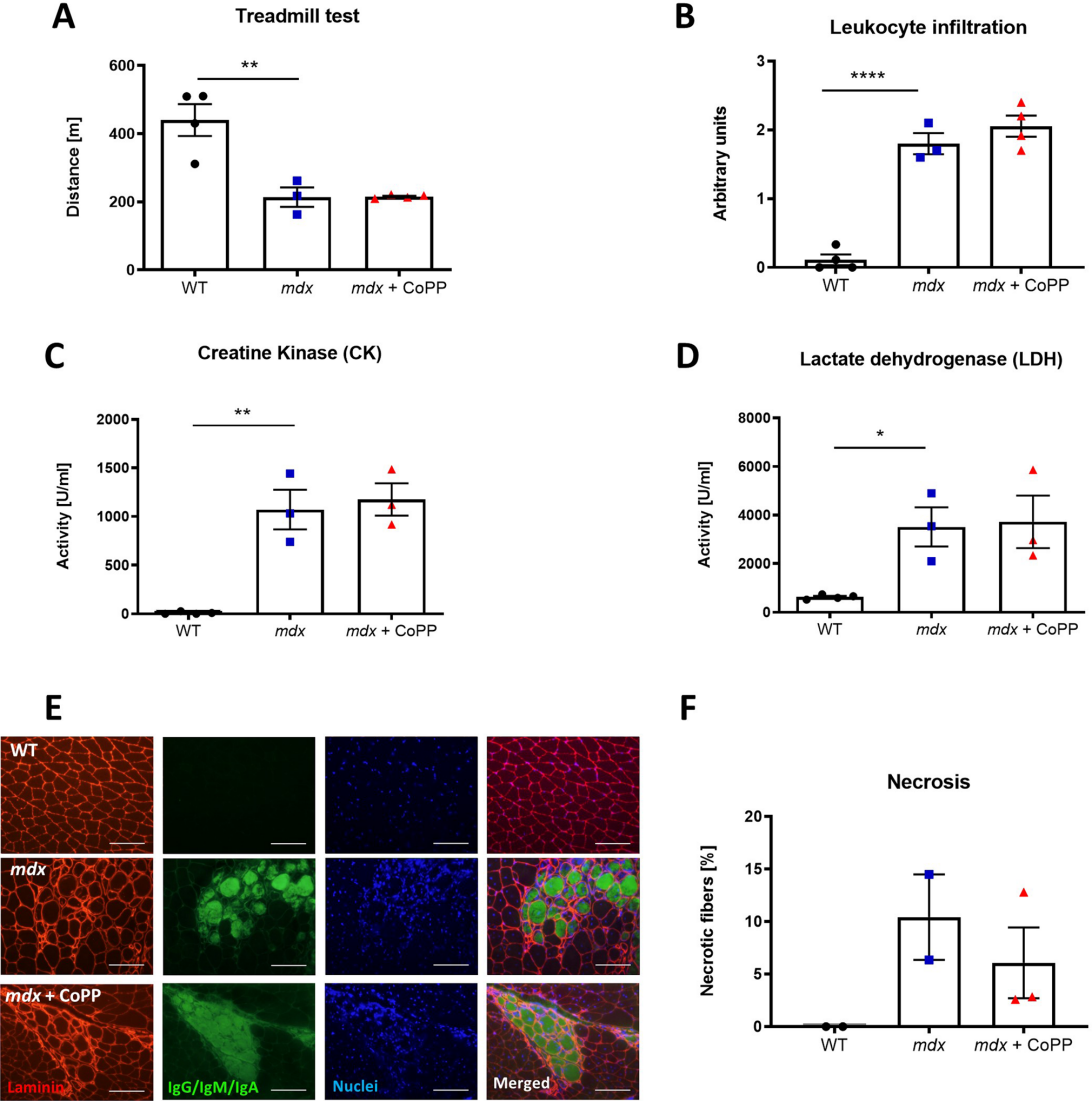
Muscle degeneration does not decrease after cobalt protoporphyrin (CoPP) treatment in dystrophic mice. **A** Running capacity of mice. Results of the treadmill test to exhaustion. **B** Semiquantitative analysis of inflammation based on hematoxylin and eosin staining in the gastrocnemius muscle. Quantitative analysis of **C** creatine kinase (CK) and **D** lactate dehydrogenase (LDH) activities in mouse plasma. Results are shown as mean ± SEM, *n* = 3–4, *p < 0.05, **p < 0.01, ****p < 0.0001; a one-way ANOVA followed by Tukey’s post-hoc test. **E**, **F** Analysis of necrosis in the gastrocnemius muscle. **E** Representative pictures of immunofluorescent staining of IgM/IgG/IGA binding (green), laminin α-2 (red), and nuclei (Hoechst, blue) in gastrocnemius muscle cross sections, scale bar = 100 μm (100 × magnification). **F** The assessment of necrotic fibers based on the performed staining, *n* = 2–3. The Kruskal–Wallis test revealed that there is no significant difference between analyzed groups

To thoroughly evaluate the impact of CoPP on disease severity, we first assessed muscle functionality by applying a downhill treadmill test. Dystrophic animals exerted a decrease in exercise capacity (*p* = 0.0038); however, in CoPP-treated *mdx* mice, no differences were observed compared to their untreated counterparts (Fig. 5A). As inflammation contributes to disease progression, H&E staining was performed and the level of leukocyte infiltration within muscle fibers was analysed. In WT mice, no infiltration was found among the myofibers, while all *mdx* mice exhibited inflammation. However, no significant differences were found between vehicle and CoPP-treated *mdx* mice (Fig. 5B). Furthermore, typical markers of DMD, CK and LDH, potently elevated in serum of *mdx* animals (*F*_2, 7_ = 25.17, *p* = 0.0006 and *F*_2, 7_ = 6,816, *p* = 0.0227, respectively) (Fig. 5C, D) were not affected by CoPP administration. Additionally, muscular necrosis, assessed by the uptake of IgG/IgM/IgA, membrane-impermeable markers [24], was intensified in *mdx* mice without any effect of CoPP treatment, as shown by the exemplary staining and its calculation (Fig. 5E, F).

### CoPP treatment has no effect on skeletal muscle fibrosis in mdx mice

**Fig. 6:**
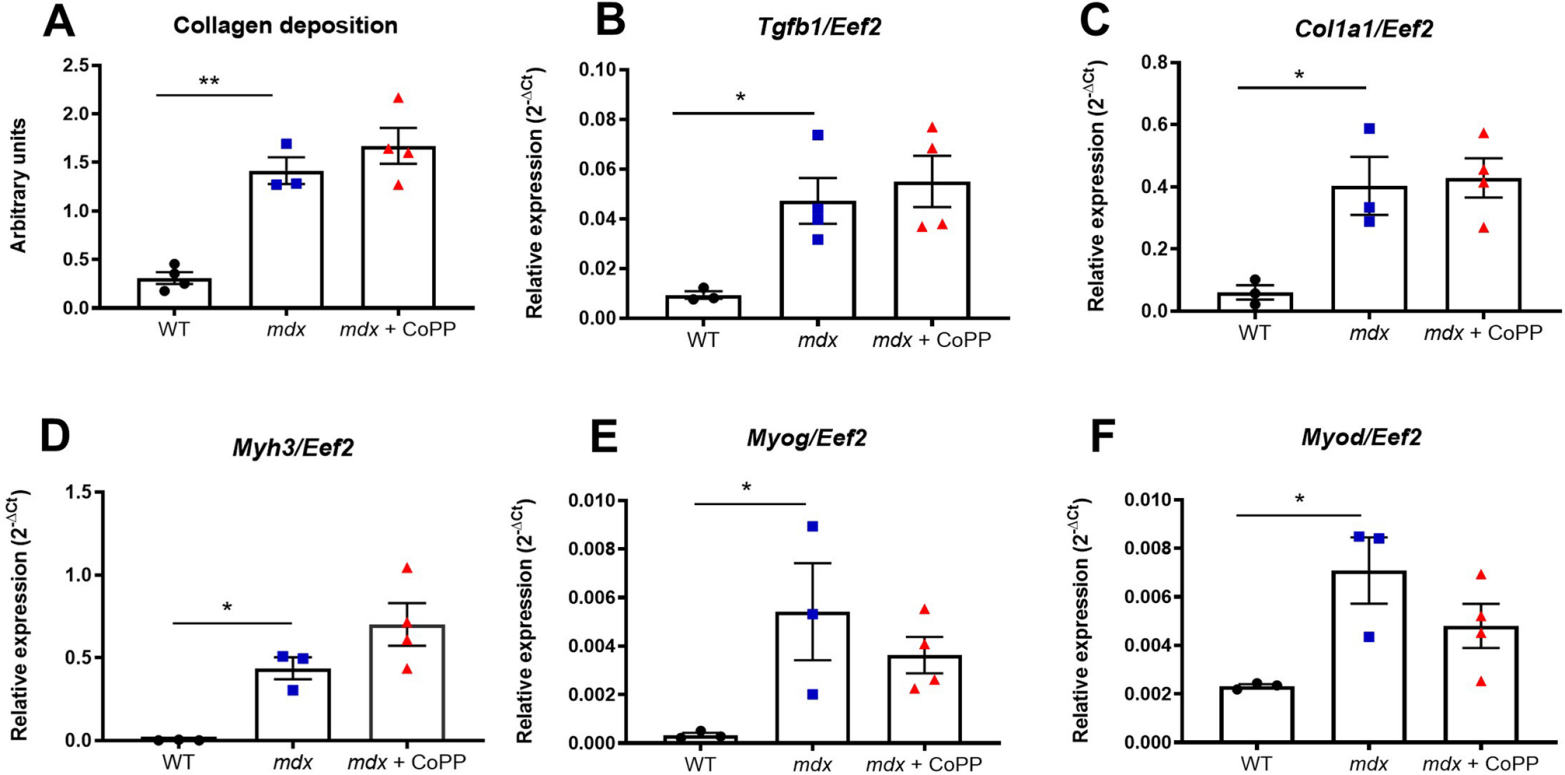
Cobalt protoporhyrin (CoPP) treatment has no effect on skeletal muscle fibrosis and muscle regeneration in *mdx* mice. **A** Semiquantitative analysis of Masson’s trichrome staining indicating increased collagen deposition in *mdx* mice without any influence of CoPP treatment. Relative expression of **B** *Tgfb1* and **C** *Col1a1* in the gastrocnemius muscle. The analysis of muscle regeneration markers: **D** *Myh3* (embryonic myosin heavy chain), **E** *Myog* (myogenin) and **F** *Myod* (myogenic determination 1) indicates no influence of CoPP treatment. Results are shown as mean ± SEM, *n* = 3–4, *p < 0.05, **p < 0.01; a one-way ANOVA followed by Tukey’s post-hoc test

To better evaluate the effect of HO-1 induction on dystrophic muscles, the impact of CoPP treatment on fibrosis was evaluated. We have observed evident collagen depositions in muscle sections of *mdx* mice, as shown by semiquantitative analysis of Masson trichrome staining (Fig. 6A). However, CoPP treatment did not attenuate fibrosis. No effect of CoPP was found when the fibrosis extent was estimated by trichrome staining for collagen (Fig. 6A) and by analysis of the expression of fibrotic markers (Fig. 6B, C). A one-way ANOVA demonstrated a statistically significant increased expression of the transforming growth factor-β (*Tgfb1)* (*F*_2, 8_ = 6.826, *p* = 0.0186) and collagen type I alpha 1 chain (*Col1a1)* (*F*_2, 7_ = 9.195, *p* = 0.0110) in dystrophic animals, but it did not indicate an effect for CoPP delivery (p = 0.7987 and p = 0.9587, respectively) (Fig. 6B, C).

### Treatment with CoPP does not influence muscle regeneration

As inflammation and fibrosis disturb muscle regeneration, we wondered if CoPP treatment can affect muscle fiber regeneration capacity. In dystrophin-lacking control animals, the expression of *Myh3*, which encodes eMHC, increased (*p* = 0.0484), but was not influenced by CoPP treatment (Fig. 6D). Furthermore, a one-way ANOVA did not demonstrate statistically significant effect of CoPP on the transcript level of other regenerative markers, *Myog* (*F*_2, 7_ = 4.552, *p* = 0.0541 and *Myod* (*F*_2, 7_ = 5.623, *p* = 0.0350) (Fig. 6E, F).

## Discussion

Muscle regeneration is a highly organized process that requires the strictly controlled cooperation of several types of cells, with the superior role of muscle satellite cells (mSCs). Disturbed regeneration is a hallmark of DMD, a genetic disorder caused by mutations in the structural protein dystrophin, which is also expressed in mSCs [25]. Based on our previous studies showing the crucial role of HO-1 in various processes that contributed to muscle pathology, including mSC differentiation [13, 18, 26], in the present work we extended our analysis by applying in vitro and in vivo models based on hiPSC-derived skeletal muscle cells overexpressing HO-1 (hiPSC-SkM-HO-1) and induction of HO-1 by CoPP treatment. We have observed that HO-1, both during human cell differentiation in vitro and after administration of HO-1 overexpressing hiPSC-SkM in vivo, partially inhibited cell differentiation. However, although in our previous study we found HO-1 deficiency as a condition that aggravates the progression of DMD [13], its strong global induction with CoPP was unable to attenuate the main hallmarks of the disease.

HO-1 can play a role in the regulation of myoblast proliferation and muscle regeneration, but this effect may depend on the duration/extent of its expression. Inhibition of myoblast differentiation after long-term expression of HO-1 was related to the regulation of MRFs (MyoD, MyoG), muscle-specific proteins (MHC), and myomiRs (miR-1, miR-133a, miR-133b, and miR-206) [26]. The results of the present study also indicate a similar effect in human muscle cells derived from hiPSCs, pointing the universality of the process, which is not only restricted to murine mSCs [13] or C2C12 myoblasts that overexpress HO-1 [26]. Accordingly, when we compared the mRNA expression of both MRFs and myomiRs between hiPSC-SkM-ctr and hiPSC-SkM-HO-1 cells we found their lower level caused by HO-1 overexpression. Furthermore, the MHC protein, a marker of mature muscle cells, was expressed at a much lower level in hiPSC-SkM-HO-1 cells than in hiPSC-derived control myotubes, suggesting the inhibitory effect of HO-1 on myogenesis. It was also the case when HO-1 was induced by CoPP. Furthermore, we observed an increase in the expression of myomiRs during satellite cell formation (days 3—8) and then during myotube formation (day 15) and the inhibitory effect of HO-1 overexpression. Accordingly, studies in murine myoblasts showed that HO-1 reduces the level of miR-1, miR-133a, miR-133b, and miR-206 by decreasing the expression of Lin28 and DGCR8 proteins necessary for the maturation of miRNA molecules [26].

Of importance, these results were also to some extent confirmed in vivo*,* as the decrease in the percentage of regenerating fibers and the expression of *Myh3*, miR-206, and the increase in *Myor* were found after the administration of HO-1 overexpressing cells to CTX-damaged muscles. MyoR, a myogenesis inhibitor, was shown to decrease after miR-206 introduction [27], and miR-206 was reduced after the administration of HO-1-overexpressing cells. Therefore, HO-1 can activate MyoR by inhibiting miR-206, which has a negative effect on differentiation. Nevertheless, lower number of centrally nucleated fibers found in animals injected with HO-1 overexpressing cells, as well as decreased expression of *Myh3* and miR-206 may indicate a more advanced stage of muscle regeneration, suggesting that hiPSC-SkM-HO-1 but not control hiPSC-SkM-ctr somehow improved regeneration.

Despite the observed influence of HO-1 overexpressing hiPSC-SkM on the regenerative potential of murine muscle, we were unable to detect human cells in mouse tissue. One month after intramuscular injection of hiPSC-SkM-ctr and hiPSC-SkM-HO-1 cells, bioluminescence imaging, flow cytometry-based analysis of the presence of cells expressing CD56 and α7integrin as well as the evaluation of the GFP signal in CD34-positive satellite cells did not demonstrate the presence of human cells in mouse muscles. This may indicate that hiPSC-SkM-HO-1 cells affected earlier stages of muscle regeneration. Problems with the survival and/or integration of myoblasts/in vivo engraftment have been shown to limit the application of this technology (for references, see: [28]). Therefore, modifications of established protocols to increase the number of myogenic progenitors and their regenerative potential in vivo seem to be crucial for a successful therapy. It was shown that the overexpression of Pax7 transcription factor [29] or the inhibition of p38 signaling pathway in vitro during mSC expansion enhances the cell potential to engraft in vivo [30]. A recent study by Choi et al. [28] suggested modulation of NOTCH and PDGF signalling as a way to increase the migration of hiPSC-derived muscle cells for more efficient muscle cell therapies. The absence of hiPSC-derived myoblasts in murine muscle tissue may be affected by the number of injected cells, their (immature) properties, or the timing of the experiment. We are aware that one of the limitations of our study is that we analysed the effect of transplantation of hiPSC-SkM from day 13 of differentiation. Further work is necessary to optimize the differentiation method to obtain a large number of cells in an earlier stage of differentiation to test their effectiveness in vivo*.* Although our results indicate that the model we used has limited potential for in vivo application, on the other hand, in our previous study, cardiomyocytes differentiated from the same hiPSCs efficiently engrafted into murine hearts and were effective in improving heart function after myocardial infarction [21].

CTX-induced acute muscle damage mimics to some extent the chronic changes seen in DMD. mSCs isolated from dystrophic *mdx* mice are characterized by reduced expression of HO-1, which results in increased differentiation [13]. Therefore, it can be suggested that up-regulation of the HO-1 level in these cells will reverse this unfavourable phenotype and thus restore the normal regenerative potential of mSCs in patients with DMD. Our research confirms that overexpression of HO-1, both during myogenic differentiation of hiPSCs in vitro and after the administration of hiPSC-derived cells in vivo, inhibits the formation of mature muscle fibers. Consequently, pharmacological treatment with HO-1 inducers, as also shown in this study with CoPP-stimulated hiPSC-derived myoblasts, confirms the hypothesis of the inhibitory effect of this enzyme on the maturation of skeletal muscle cells.

However, despite the previously observed aggravation of the disease in *mdx* animals devoid of HO-1 [13], our current results did not reveal the beneficial effect of CoPP-induced HO-1 up-regulation in dystrophic animals. HO-1 has been proposed as a target for DMD treatment, primarily due to its anti-inflammatory and cytoprotective functions, which derive from the degradation of pro-oxidant heme and the important roles of its metabolites [31, 32]. HO-1 is a crucial agent in processes related to the pathophysiology of DMD and can regulate muscle damage, inflammation, and regeneration [13, 18]. However, there is disagreement about the expression of HO-1 in the whole muscle tissue of dystrophic mice, as its down-regulation [19, 33] and up-regulation have been evident [13, 34]. However, in contrast to the whole muscle, the expression of this cytoprotective enzyme was much lower in mSCs isolated from *mdx* mice than from wild-type animals, which contributed to their increased differentiation [13]. Despite the different regulation in dystrophic muscles and mSCs, HO-1 alleviated the progression of DMD [13].

In the present work, the efficacy of CoPP stimulation was confirmed by up-regulation of HO-1 at mRNA and protein levels, both in the liver and in skeletal muscles, compared to vehicle-treated *mdx* mice. However, this induction of HO-1 expression did not result in attenuation of dystrophic muscle pathology. In our study, the administration of CoPP influenced neither exercise capacity, inflammation, fibrosis, nor the regenerative potential of dystrophic muscles, while we previously demonstrated that genetic loss of the HO-1 gene resulted in lower running performance in the treadmill test, higher levels of inflammation, and altered regeneration [13]. In contrast, Chan et al. showed that CoPP treatment reduced muscle damage in *mdx* mice [19]. The differences between these studies may result from subtle technical variables. Although the dose and weekly interval of CoPP administration were the same, Chan et al. obtained a three-fold induction in the level of *Hmox1* mRNA in the gastrocnemius muscle, while in our hands, the level of HO-1 increased about 10 times. Noteworthy, high HO-1 overexpression can have detrimental effects. Suttner and Dennery suggested that low HO-1 overexpression exerts cytoprotective activity, but its exaggerated level can lead to an accumulation of reactive iron, resulting in increased oxidative stress and cytotoxicity, along with abnormal cell proliferation [35]. We also previously demonstrated a dual effect of CoPP on kidney injury, when moderate induction of HO-1 attenuated nephrotoxicity, while its very high up-regulation resulted in the profibrotic and pro-apoptotic effect [36]. Additionally, we found abnormal cellular proliferation and greater tumorigenic potential in myoblasts overexpressing HO-1 [26].

### Study limitations and future perspectives

First, our study was limited to analysis of the delivery of hiPSC-SkM to the anterior tibialis and the effect of CoPP in the gastrocnemius muscles, while both muscles may differ in the proportion of slow and fast twitch fibres and general metabolism. Second, we have used dystrophic animals to study the effect of CoPP-induced HO-1 overexpression on the regeneration potential. In *mdx* mice, continuous overlapping cycles of damage and regeneration occur, while other models of muscle injury using myotoxic agents, such as cardiotoxin (CTX) and notexin (NTX), chemical compounds (barium chloride and glycerol), and physical approaches (crush, denervation, and freeze injury) can differ in the course of regeneration. Therefore, we cannot exclude the possibility that the effects of CoPP will depend on the approach used. Third, we evaluated the effect of transplantation of hiPSC-SkM at specific day of differentiation and more experiments should be performed to assess the effect of such cells obtained at an earlier stage of differentiation to test their effectiveness in vivo*.* Finally, this analysis only includes selected regeneration markers and does not evaluate, for example, Pax3, MRF4 and other factors that play an important role in the regulation of muscle regenerative potential. Therefore, the continuation of this topic should be addressed in the future.

## Conclusions

We have demonstrated that the global induction of HO-1 expression by CoPP treatment, while potentially increasing HO-1 level in satellite cells, could overregulate it too strongly in skeletal muscles. We suppose that this may not be beneficial and propose that specifically targeting and regulating HO-1 expression might be necessary for its protective effect in DMD. Overall, we assume that HO-1 may exert a protective effect in the strictly defined therapeutic window and its induction for therapeutic purposes has to be controlled. Additional research is needed to fully determine the validity of CoPP as a potential therapeutic agent for DMD.

## Supplementary Information

Below is the link to the electronic supplementary material.

Supplementary file1 (DOCX 40 KB)

Supplementary file2 (PDF 1142 KB)

## Data Availability

The datasets generated during and/or analyzed during the current study are available from the corresponding authors upon reasonable request.

## Abbreviations

CK: Creatine kinase
Col1a1: Collagen type I alpha 1 chain
CoPP: Cobalt protoporphyrin IX
CTX: Cardiotoxin
DMD: Duchenne muscular dystrophy
Hmox1/HMOX1: Gene encoding HO-1
HO-1: Heme oxygenase-1
hiPSC: Human induced pluripotent stem cell
hiPSC-SkM: Human induced pluripotent stem cell-derived skeletal muscle cells
hiPSC-ctr: Control hiPSC
hiPSC-HO-1: HiPSC overexpressing heme oxygenase-1
LDH: Lactate dehydrogenase
MRFs: Myogenic regulatory factors
mSCs: Muscle satellite cells
Myf5: Myogenic factor 5
MHC: Myosin heavy chain
Myh3: Myosin heavy chain 3
MyoD: Myogenic determination factor
MyoG: Myogenin
NRF2: Nuclear factor erythroid 2-related factor 2
Pax7: Paired box
Tgfb1: Transforming growth factor-β
