# Supplementary material for: Effect of heme oxygenase-1 on the differentiation of human myoblasts and the regeneration of murine skeletal muscles after acute and chronic injury
Source: Pharmacol Rep. 2023 Mar 15;75(2):397–410. doi: 10.1007/s43440-023-00475-3 (PMC10060298; doi:10.1007/s43440-023-00475-3)
Supplement: Supplementary file 1 — Supplementary file1 (DOCX 40 KB) [file 43440_2023_475_MOESM1_ESM.docx]

Supplementary Materials and Methods:

Effect of heme oxygenase-1 on the differentiation of human myoblasts

and the regeneration of murine skeletal muscles after acute and chronic injury

Urszula Głowniak-Kwitek, Asier Laria Caballero, Iwona Bronisz-Budzyńska, Magdalena Kozakowska, Kalina Andrysiak, Jacek Stępniewski, Agnieszka Łoboda^*^, Józef Dulak^*^

Department of Medical Biotechnology, Faculty of Biochemistry, Biophysics and Biotechnology, Jagiellonian University, 30-387 Kraków, Poland;

* Correspondence: agnieszka.loboda@uj.edu.pl; Tel.: +48-12-664-6412; jozef.dulak@uj.edu.pl; Tel.: +48-12-664-6398

Generation and differentiation of human induced pluripotent stem cells

The hiPSCs line was obtained by transduction with Sendai vectors (CytoTune-iPS 2.0 Sendai Reprogramming kit) of peripheral blood mononuclear cells (PBMC) obtained from a healthy donor. The presence of markers characteristic for pluripotent stem cells: OCT4, NANOG, SSEA4, TRA-1-60 and TRA-1-81, as well as the ability to differentiate *in vitro* to cells derived from three germ layers was confirmed in our previous study [21]. To obtain modified hiPSC lines, cells were transduced with lentiviral vectors encoding green fluorescent protein (GFP), luciferase (Luc), and heme oxygenase-1 (HO-1). The resulting control (hiPSC-ctr) and HO-1 overexpressing (hiPSC-HO-1) hiPSC lines were purified using a cell sorter (Fluorescence Activated Cell Sorting, MoFlo XDP Cell Sorter, Beckman) based on the GFP signal as previously described [21]. They were further cultured in an incubator (37°C, 5% CO_2,_ 95% humidity) in the Essential 8 medium (Thermo Fisher Scientific). For cell passage, EDTA (0.5 mM in PBS ion-free, PAA) was used. Expression of luciferase in hiPSC-derived cells was then used for *in vivo* imaging. The GFP level was analysed by flow cytometry.

**RNA isolation, reverse transcription, and quantitative real-time PCR (qRT-PCR)**

For RNA isolation, hiPSC-SkM growing in a 24-well plate were trypsinized, centrifuged and the cell pellet was dissolved in 400 µl fenozol (A&A Biotechnology). For RNA isolation from mouse tissues, a piece of liver, gastrocnemius, and tibialis anterior muscle was collected, preserved in RNAlater RNA Stabilization Solution (Sigma-Aldrich), snap-frozen in liquid nitrogen, and stored at -80°C until processed. RNA isolation was performed following the QIAzol lysis reagent protocol (Qiagen), and subsequent homogenization in a TissueLyser mechanical disruptor (Qiagen) based on the standard Chomczynski-Sacchi method [22] and phenol-chloroform extraction as described in our previous study [13]. The concentration and quality of RNA were determined using a Nanodrop 1000 spectrophotometer (Thermo Fisher Scientific). cDNA templates were synthesized from 1 μg of total RNA diluted in RNase-free water (Polpharma) with recombinant M-MuLV reverse transcriptase (Thermo Fisher Scientific), oligo(dT) primers and dNTPs (both from Genomed). The miRCURY LNA RT Kit (Qiagen) or MystiCq microRNA cDNA synthesis (Sigma-Aldrich) were used to check the expression of miR-133a and miR-133b or miR-1 and miR-206, respectively. To perform qRT-PCR, the mixture containing the SYBR Green PCR Master Mix (Sigma-Aldrich), specific primers (Table 1; sequences for miR-133a and miR-133b were provided by vendor), and cDNA was prepared in a 96-well plate and analysed in the StepOnePlus Real-Time PCR system (Applied Biosystems). For negative controls, nuclease-free water was used instead of cDNA. Gene expression levels were calculated by normalizing to the level of the housekeeping gene encoding elongation factor 2 (*Eef2/EEF2*) or the constitutive small nuclear RNA U6 (U6 snRNA) in the case of microRNAs. The relative expression level was calculated as 2^-ΔCt^ values, where ΔCt = C_t gene of interest_ – C_t_ *_Eef2/EEF2/U6_* _snRNA_). Primer specificity was monitored based on the melting curves.

**Table 1**: Sequences of primers used for qPCR analysis.

**Western blotting**

The tissues were homogenized using TissueLyser (Qiagen) in PBS containing 1% Triton X-100 (BioShop) and protease inhibitors (cOmplete^TM^, Mini Protease Inhibitor Cocktail; Roche Diagnostic) and centrifuged (14 000 × *g*for 10 min at 4°C). The pellets from cells were prepared in the same way. The total protein content was evaluated using the bicinchoninic acid assay (BCA) (Sigma-Aldrich). For the evaluation of the protein level of HO-1, GAPDH and α-tubulin, 25 µg of protein lysates were subjected to SDS-PAGE electrophoresis, transferred to a nitrocellulose membrane, blocked in 5% non-fat milk in TBS with 0.1% Tween20 (blocking buffer, BioShop) (1 hour, room temperature), and incubated overnight at 4°C with primary antibodies: mouse anti-α-tubulin (T9026, clone DM1A, Sigma-Aldrich) and rabbit anti-HO-1 (ADI-SPA-894-F, Enzo), diluted 1:1000 in blocking buffer. The following day, the membranes were washed 5 times in TBS with 0.1% Tween for 5 min, and subsequently incubated with HRP-linked secondary antibodies: goat anti-mouse for α-tubulin (BD Biosciences) and goat anti-rabbit for HO-1 (Cell Signalling) diluted 1:10,000 in blocking buffer for 1 hour at room temperature. The membranes were washed again 5 times for 5 min in TBS with 0.1% Tween. The Immobilon Western Chemiluminescent HRP Substrate (Millipore) was added to the membrane to obtain luminescent signals and bands for protein detection were developed using an X-ray film (AGFA). GAPDH (1:1000, sc-59540, Santa Cruz Biotechnology) was developed after stripping the membrane from the initial antibodies by two washings with 0.1M glycine (7.5 g/l, pH 2.6) for 30 min at room temperature.

**Histological analyses**

The gastrocnemius muscles were placed in 10% formalin for 48 hours. Subsequently, the tissues were processed in an Excelsior ES tissue processor. The muscles were embedded in paraffin blocks in a HistoStar embedding station and cut at 4 μm sections using a Microm H355S microtome, and placed on poly-L-lysine covered glass slides. After deparaffinization, the sections were subjected to hematoxylin and eosin (H&E) and Masson’s trichrome stainings according to the vendor’s instructions (Sigma-Aldrich). Deparaffinization and histological staining were performed in a Varistain Gemini tissue staining station (all apparatus from Thermo Fisher Scientific). The degree of inflammation was evaluated based on H&E staining according to a semi-quantitative 0-4 scale (0 = no signs of inflammation; 1 = minor signs of leukocyte infiltration; 2 = visible inflammation; 3 = signs of inflammation found in the half of the image; 4 = high rate of inflammatory cell infiltration, inflammation present around the whole image). The sections stained with H&E were also used for the analysis of regeneration extent (assessed based on the presence of centrally nucleated myofibers and shown as the percentage of all fibers). The level of collagen deposition was evaluated after Masson’s trichrome staining using the 0-4 scale (0 = no signs of collagen deposition; 1 = minor signs of collagen deposition; 2 = visible collagen deposition; 3 = signs of collagen deposition present in about half of the image; 4 = collagen deposition present throughout the image). All analyses were performed by an observer blinded to the treatment of animals, and 10 images at 100x magnification of muscle sections per mouse were analysed *via* ImageJ software.

**Immunohistofluorescence staining**

For the preparation of frozen sections, gastrocnemius muscles were harvested and snap-frozen in OCT Tissue Freezing Medium (Leica) in a pre-chilled isopentane bath cooled with liquid nitrogen. The samples were stored at -80°C until use. Cross-sections were obtained by cutting 10 μm slides from the frozen muscles in a cryostat (Leica CM1850) and placing them on poly-L-lysine covered glass slides. For evaluation of necrotic fibers (accumulation of IgG, IgA, and IgM antibodies), air-dried for 45 min frozen sections were blocked with 10% goat serum (Sigma-Aldrich) in PBS for 1 hour in RT, washed in PBS, incubated with primary rabbit anti-mouse laminin α-2 (1:1000 dilution, 4H8-2, Sigma-Aldrich) at 37°C for 1 hour and washed in PBS. The goat anti-mouse IgG/IgA/IgM Alexa Fluor 488 (for the detection of necrotic fibers) (1:50 dilution, Thermo Fisher Scientific), goat anti-rabbit, Alexa Fluor 568 (for the detection of laminin α-2) (1:500 dilution, Thermo Fisher Scientific) antibodies were then added for 1 hour at 37°C. The slides were then washed once for 5 min and the nuclei were stained with Hoechst (1:10,000 dilution, 2 µg/ml, Sigma-Aldrich). The staining was analysed by ImageJ software. To assess necrosis in the damaged area, the percentage of necrotic myofibers (with increased uptake of IgG/IgM/IgA) was counted among the total number of fibers, in 10 pictures per muscle at 100x magnification.
