# Supplementary material for: Effect of heme oxygenase-1 on the differentiation of human myoblasts and the regeneration of murine skeletal muscles after acute and chronic injury
Source: Pharmacol Rep. 2023 Mar 15;75(2):397–410. doi: 10.1007/s43440-023-00475-3 (PMC10060298; doi:10.1007/s43440-023-00475-3)
Supplement: Supplementary file 2 — Supplementary file2 (PDF 1142 KB) [file 43440_2023_475_MOESM2_ESM.pdf]

Generation of skeletal muscle from human induced pluripotent stem cells

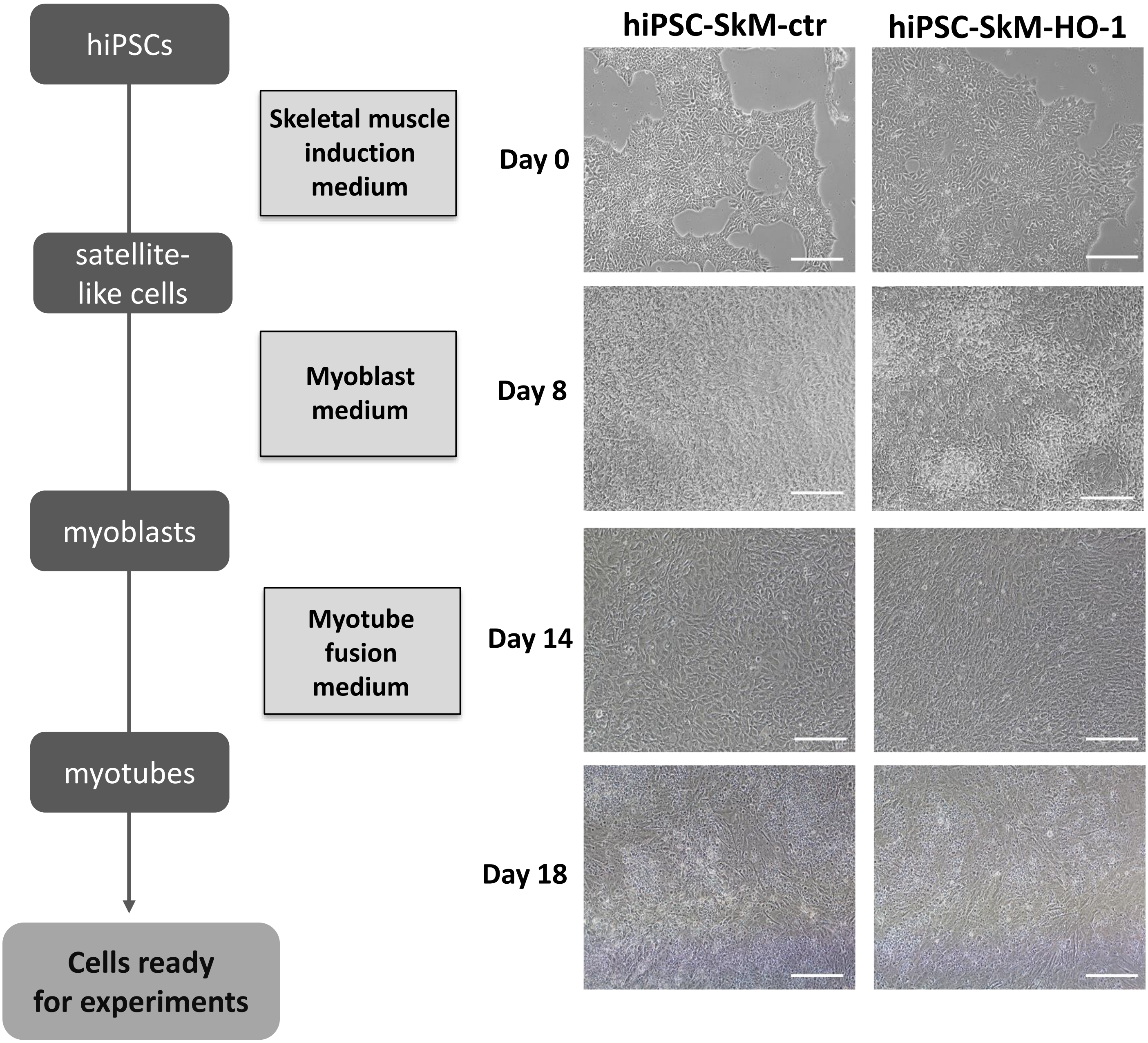

Figure S1. The general overview of the generation of skeletal muscle cells from human induced pluripotent stem cells (hiPSC-SkM). Pictures of muscle cells derived from control hiPSC and hiPSC-HO-1 were taken on days 0, 8, 14 and 18 of differentiation. Representative pictures from phase contrast microscopy, scale bar = 100 μm (100x magnification).

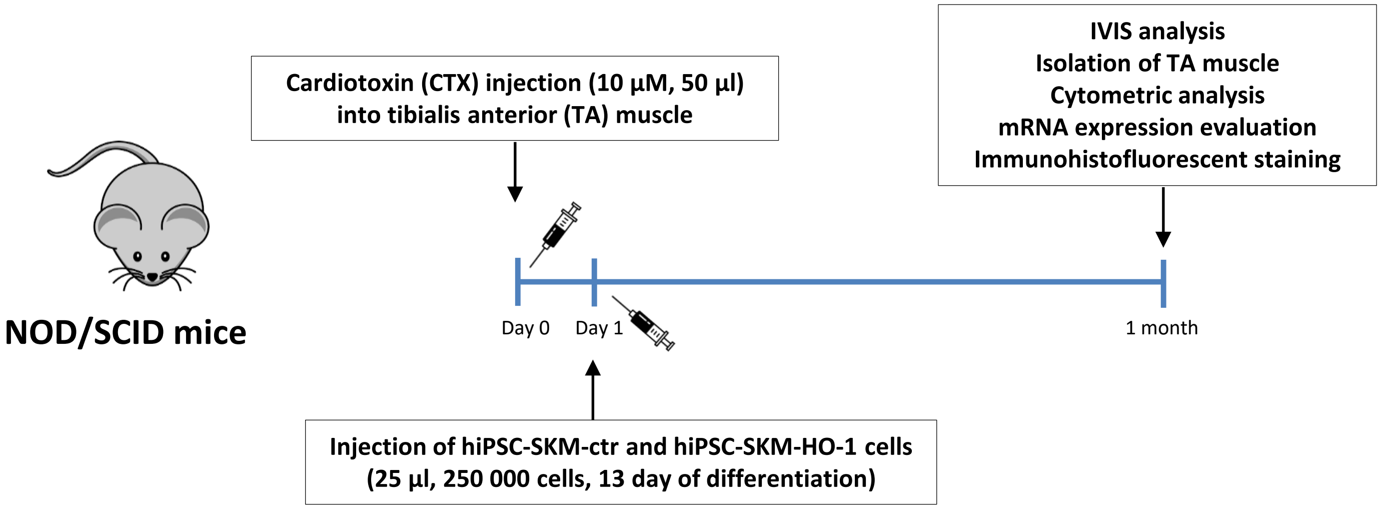

Figure S2. Scheme of hiPSC-SkM delivery to NOD/SCID mice to assess muscle regeneration *in vivo*.

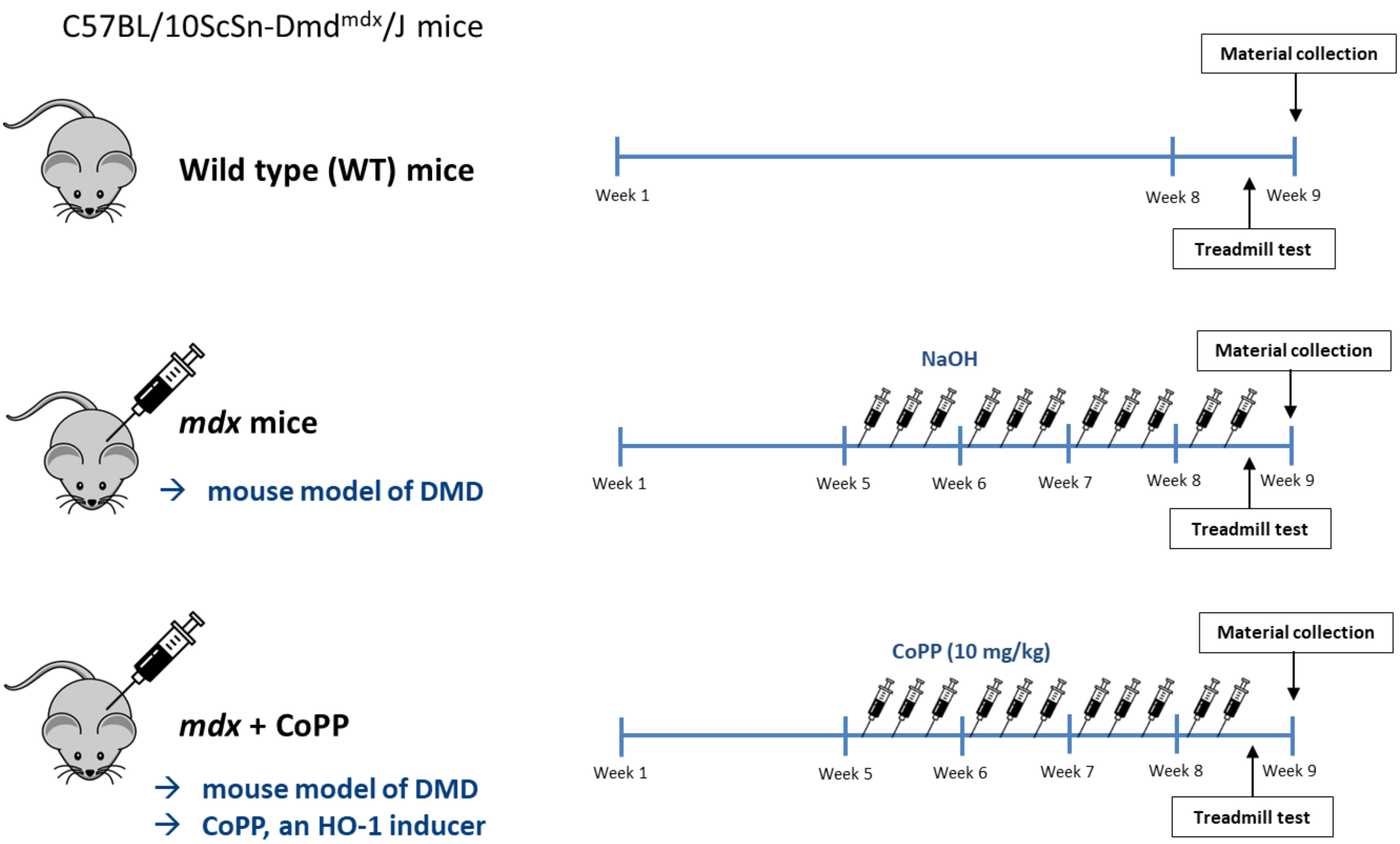

Figure S3. Setup of experiment with cobalt protoporhyrin (CoPP) injection into dystrophic *mdx* mice.

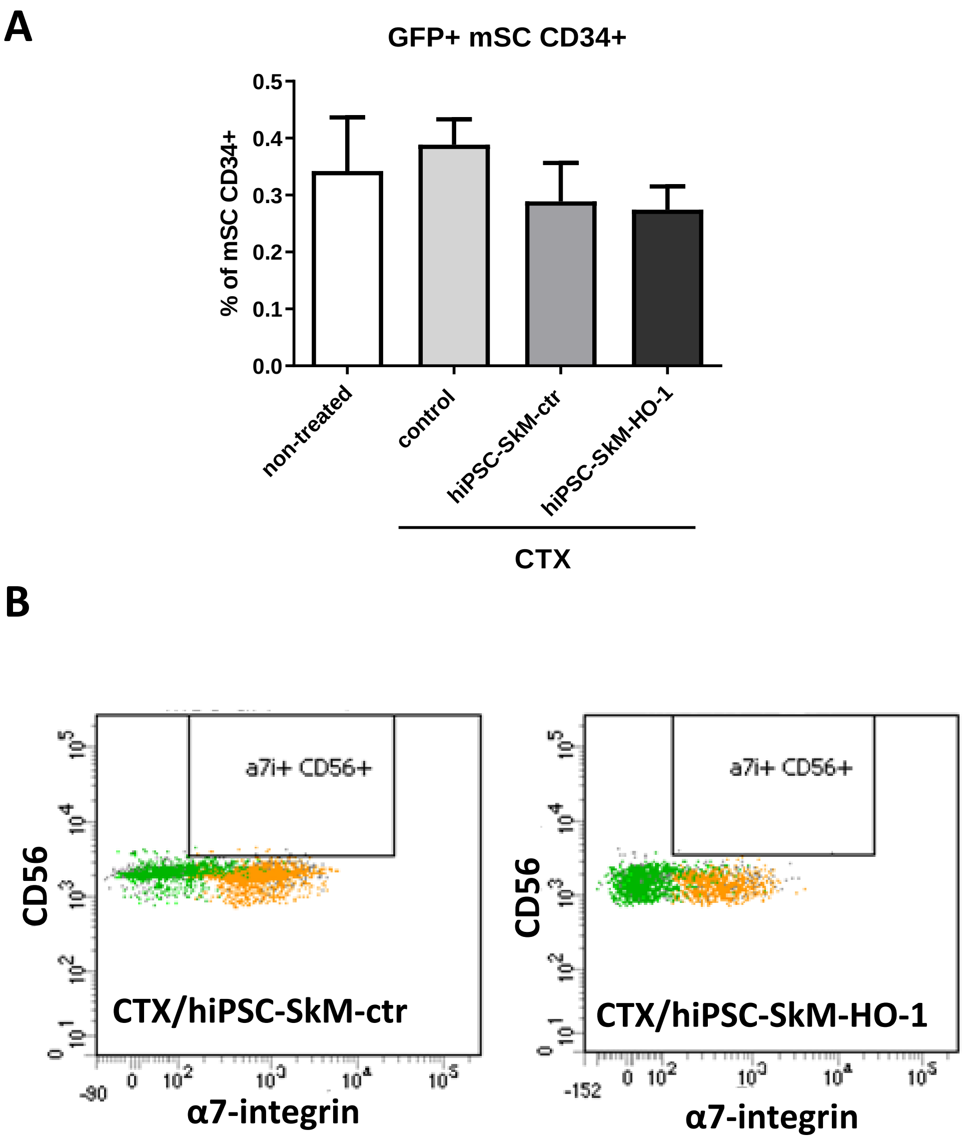

Figure S4. Analysis of the integration of human myoblasts into the muscles of NOD/SCID mice. Cytometric analysis of **(A)** $CD34^+$/GFP^+ mSCs and **(B)** $CD56^+$/α7integrin$^+$ hiPSC-SkM-ctr and hiPSC-SkM-HO-1 injected into CTX-injured tibialis anterior muscle. Results are shown as mean +/- SEM, n = 4-6.

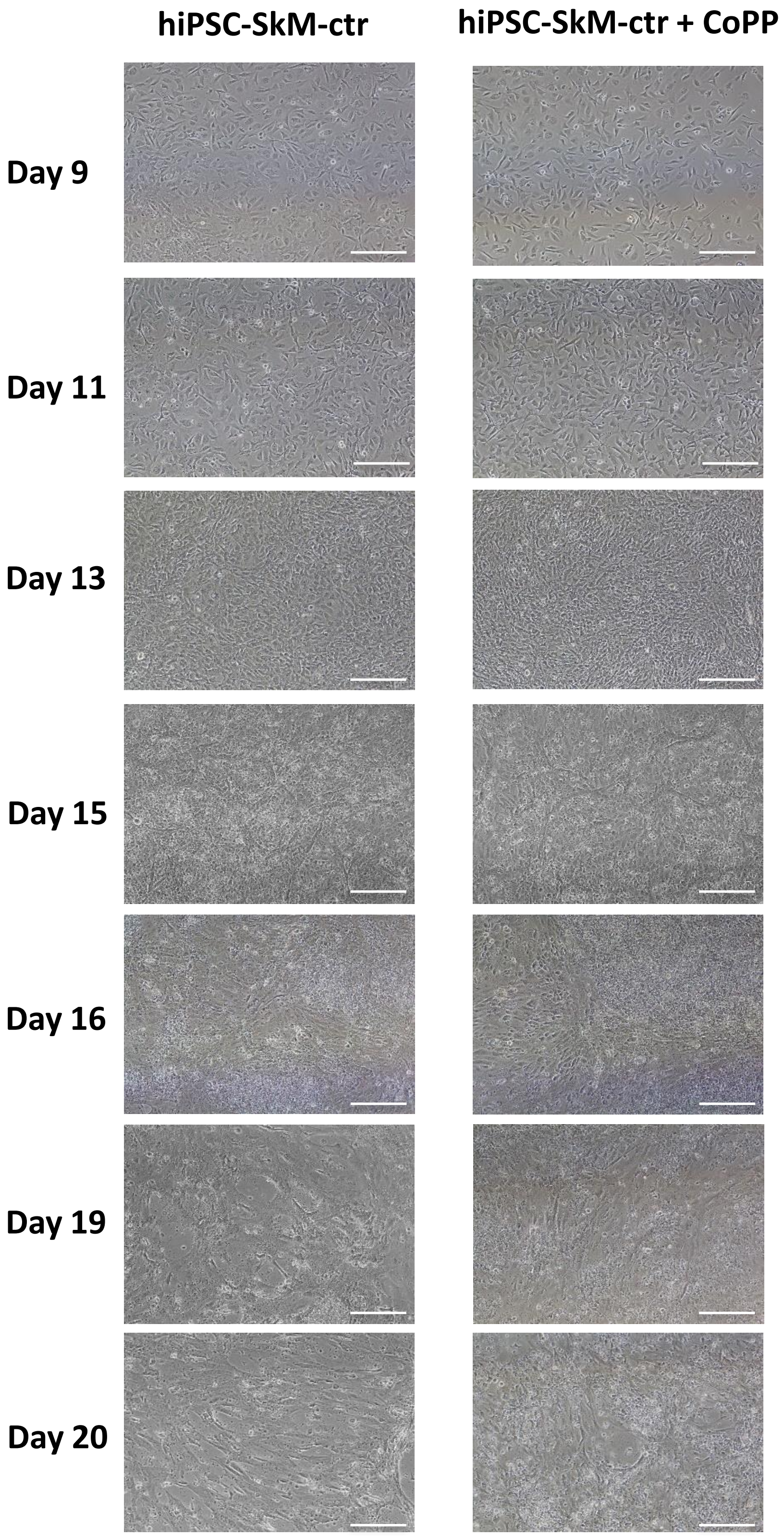

Figure S5. **The morphology of control hiPSC-SkM (hiPSC-SkM-ctr) and hiPSC-SkM-ctr treated with CoPP in the following days of differentiation process.** Representative pictures from phase contrast microscopy, scale bar = 100 μm (100x magnification).
